# Supplementary material for: Profiling of Seed Proteome in Pea (Pisum sativum L.) Lines Characterized with High and Low Responsivity to Combined Inoculation with Nodule Bacteria and Arbuscular Mycorrhizal Fungi
Source: Molecules. 2019 Apr 23;24(8):1603. doi: 10.3390/molecules24081603 (PMC6514749; doi:10.3390/molecules24081603)
Supplement: Supplementary file 1 [file molecules-24-01603-s001.zip › 190413_Mamontova_et_al_Supplementary_Information_1_Revized.pdf]

Profiling of seed proteome in pea (*Pisum sativum* L.) lines characterized with a high and low responsivity to combined inoculation with nodule bacteria and arbuscular mycorrhizal fungi

Supplementary information 1

Tatiana Mamontova[1,2§], Alexey M. Afonin[3§], Christian Ihling[4], Alena Soboleva[1,2], Elena Lukasheva[1], Anton S. Sulima[3], Oksana Y. Shtark[3], Gulnara A. Akhtemova[3], Maria N. Povydysh[5], Andrea Sinz[4], Andrej Frolov[1,2*], Vladimir A. Zhukov[3], and Igor A. Tikhonovich[3,6*]

[1]Department of Biochemistry, St. Petersburg State University, [2]Department of Bioorganic Chemistry, Leibniz Institute of Plant Biochemistry, [3]Department of Biotechnology, All-Russia Research Institute for Agricultural Microbiology, [4]Department of Pharmaceutical Chemistry and Bioanalytics, Institute of Pharmacy, Martin-Luther Universität Halle-Wittenberg, [5]R&D department, Saint-Petersburg State Chemical and Pharmaceutical University, [6]Department of Genetics and Biotechnology, St. Petersburg State University

§ These authors contributed equally to the manuscript

*Corresponding author:

Acad. Dr. Igor A. Tikhonovich
All-Russia Research Institute for Agricultural Microbiology
Department of Biotechnology
Podbelsky chaussee, 3, Pushkin, 196608,
St. Petersburg, Russian Federation
Tel. +7 (812) 4705100
Fax. +7 (812) 4704362
Email: arriam2008@yandex.ru

Dr. Andrej Frolov
Leibniz Institute of Plant Biochemistry
Department of Bioorganic Chemistry
Weinberg 3, 06120 Halle (Saale)
Germany
Tel. +49 (345) 55821350
Fax. +49 (345) 55821309
Email: afrolov@ipb-halle.de

Directory

Tables...3

Table S1-1 Instrument settings applied for ESI-Q-Orbitrap-MS experiments.........................3

Table S1-2 Result filters, set in Progenesis QIP software for processing of spectra, acquired for differentially expressed tryptic peptides, as well as the filters for corresponding proteins.. 5

Table S1-3 Individual sequence databases comprising a combined non-redundant database used for SEQUEST search engine 6

Table S1-4 Settings for the database search with SEQUEST engine.......................................7

Table S1-5 Protein recoveries and total densities calculated for individual samples separated by SDS-PAGE...8

Figures..9

Figure S1-1 Independent reproduction of the experiment presented in Figure 1......................9

Figure S1-2 Tandem mass spectra of the *m/z*, corresponding to the peptides identified in a search, performed against a redundant legume sequence database...10

Figure S1-3 Post-processing of the label-free quantification data with Perseus 1.6.0.0 software. 22

Figure S1-4 Loading plots for the principal component analysis...23

Figure S1-5 Mass spectra and extracted ion chromatograms (left and right panels, respectively) obtained Progenesis QIP 4.0 software upon quantification procedure...............24

References 26

Tables

Table S1-1 Instrument settings applied for ESI-Q-Orbitrap-MS experiments

Parameter	Setting
MS conditions	
Ionization mode	Positive
ESI emitter	outer/internal diameter 360/30 µm, 40 mm long (Thermo Fisher Scientific, Bremen, Germany)
Analyzer	Orbitrap
Resolution	120000
Ion spray voltage (IS)	1900 V
S-lens voltage	20 V
Ion transfer tube temperature	275 °C
Automatic gain control (AGC) target	$2*10^5$
Maximum injection time	50 ms
Mass to charge ratio (*m/z*) range	300 – 1500
MS/MS conditions	
Fragmentation	Collision activated dissociation
Analyzer	Orbitrap
Resolution	15000
Isolation width	2.0 *m/z*
Charge state rejected	1+, > 6+
Fragmentation	HCD
HCD collision energy	27%
Automatic gain control (AGC) target	$5*10^4$
Maximum injection time	200 ms

Dynamic exclusion repeat count	1
Dynamic exclusion duration	60 s
Dynamic exclusion mass width	± 2 ppm

Table S1-2 Result filters, set in Progenesis QIP software for processing of spectra, acquired for differentially expressed tryptic peptides, as well as the filters for corresponding proteins

Nr	Spectral filters	Peptide filters	Protein filters
1	Charge 2-6	Show ANOVA $p \leq 0.05$	Show $q \leq 0.05$
2	Rank>4	Show CV $\leq$ 60	Show FC $\geq$ 1.5 for at least one group comparison
3		Hide No MS/MS data	

Table S1-3 Individual sequence databases comprising a combined non-redundant database used for SEQUEST search engine

Nr	Species	Sequences
1	*Medicago truncatula* Gaertn	From Uniprot,01.06.2017
		From MTGD ProteinSeq v4.2, 18.08.2014
2	*Lotus japonicas* (Regel) K. Larsen	From Uniprot, 01.06.2017
		From miyakogusa.jp, 01.06.2017
		From miyakogusa.jp, 08.07.2017
3	*Phaseolus vulgaris* L	From Uniprot, 16.03.2017

All individual proteomes were fused to obtain one fasta file. The sequence redundancy was eliminated by database clustering using CD-HIT algorithm was used with sequence identity cut-off set to 1 [1]. SEQUEST search engine is implemented in Proteome Discoverer 2.1

Table S1-4 Settings for the database search with SEQUEST engine

Database search settings	
Analysis program	SEQUEST settings
Protease	Trypsin
Missed cleavage sites	2
Peptide tolerance	10 ppm
MS/MS	0.2 Da
Target FDR	5%
Signification threshold	$p \leq 0.05$
Modification	Mass increment (Da)/amino acids
Carbamidomethyl	57.021/C[a]
Oxidation	15.995/M/W

Table S1-5 Protein recoveries and total densities calculated for individual samples separated by SDS-PAGE

Sample	Weight (mg)	Concentration (mg/mL)	Recovery (mg/g fresh weight)	Average intensity
K-8274 BSM-1	49.0	61.8	126.1	13846
K-8274 BSM-2	48.3	74.4	154.0	20895
K-8274 BSM-3	51.7	66.7	129.0	19600
K-8274 NI-1	52.4	84.5	161.3	14049
K-8274 NI-2	51.5	45.1	87.6	17412
K-8274 NI-3	52.0	64.9	124.8	18800
K-3358 BSM-1	50.4	88.2	175.0	19358
K-3358 BSM--2	50.3	81.9	162.8	15780
K-3358 BSM-3	56.2	81.0	144.1	18900
K-3358 NI-1	50.9	70.8	139.1	19665
K-3358 NI-2	48.4	30.8	63.6	16900
K-3358 NI-3	50.0	51.8	103.6	17500

Average intensities were calculated in Quantity One® 1-D analysis software and normalized according to comparison average intensity of protein markers lane presented on each gel; BSM, plants, inoculated with beneficial soil microorganisms – rhizobia and AM fungi; NI, not inoculated plants

Figures

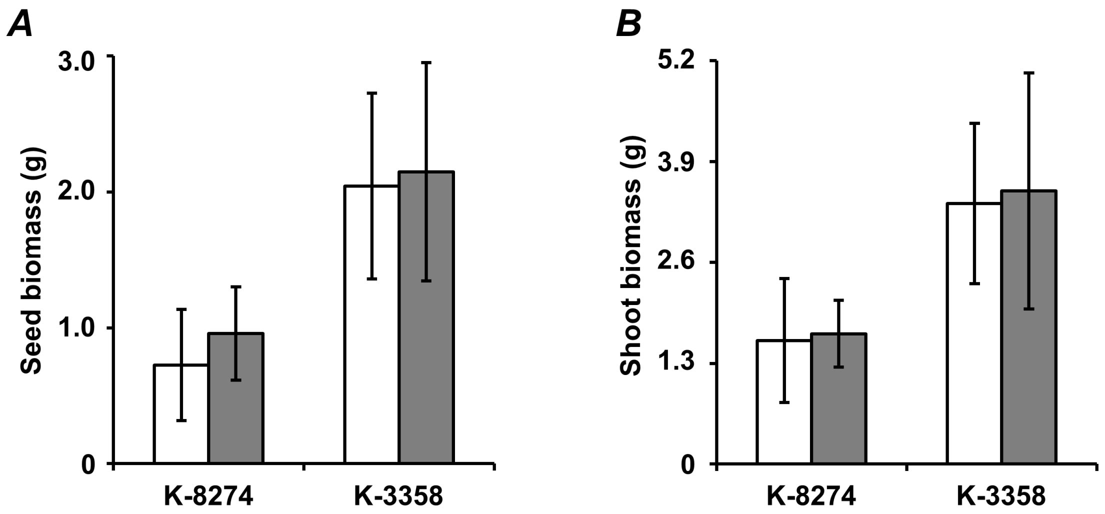

Figure S1-1 Independent reproduction of the experiment presented in Figure 1. Seed (A) and shoot (B) biomass (n = 14 for BSM inoculated groups, 9 for K-8174 NI group, 13 for K-3358 NI group) determined for the pea (*P. sativum* L) plants of the lines K-8274 (high EIBSM) and K-3358 (low EIBSM), grown with (grey) and without (white) simultaneous colonization of pea roots with rhizobia and arbuscular mycorrhizae (AM) fungi *R. irregularis*. The t-test *p* values are 0.16 and 0.73 for the seeds and 0.73 and 0.75 for the shoots upon inoculation of plant of lines K-8274 and K-3358, respectively.

Figure S1-2 Tandem mass spectra of the *m/z*, corresponding to the peptides identified in a search, performed against a redundant legume sequence database. Spectra are given in order corresponding to the order of proteins in Table 2. Number of peptide spectrum matches (PSM) was calculated by Sequest engine in Proteome Discoverer 2.2 software. M_{Ox}, methionine sulfoxide; W_{Ox}, 5-hydroxytryptophan.

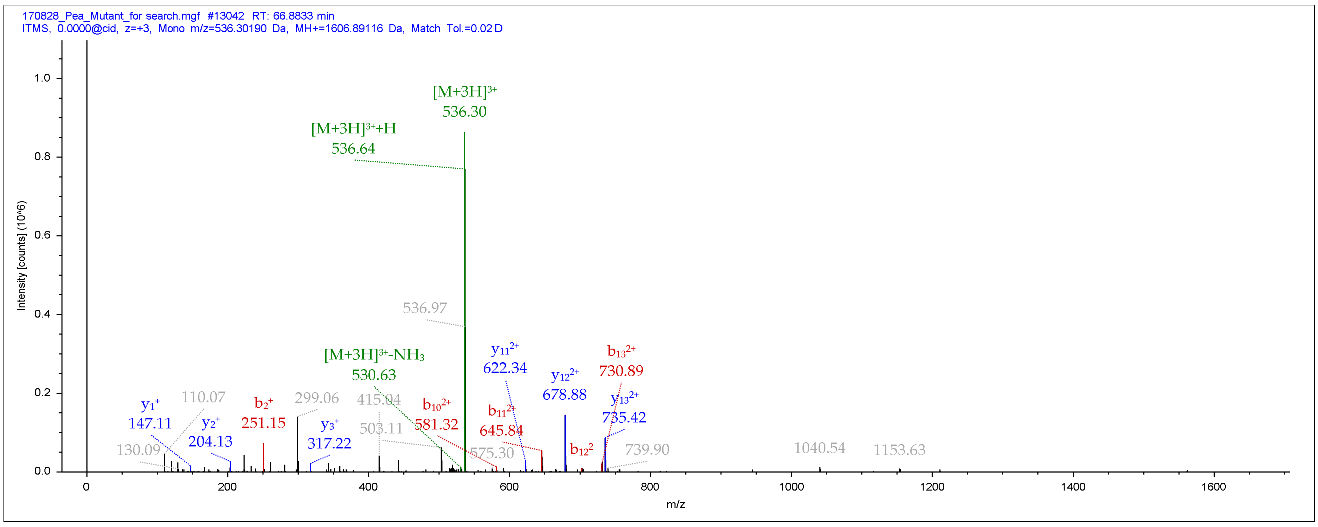

Figure S1-2.1 Q1S053 (1 unique peptide(s), master protein)

HLLLAVRNDEELGK, (1606.89116 Da, *m/z* 536.30, $[M+3H]^{3+}$), 2 PSM

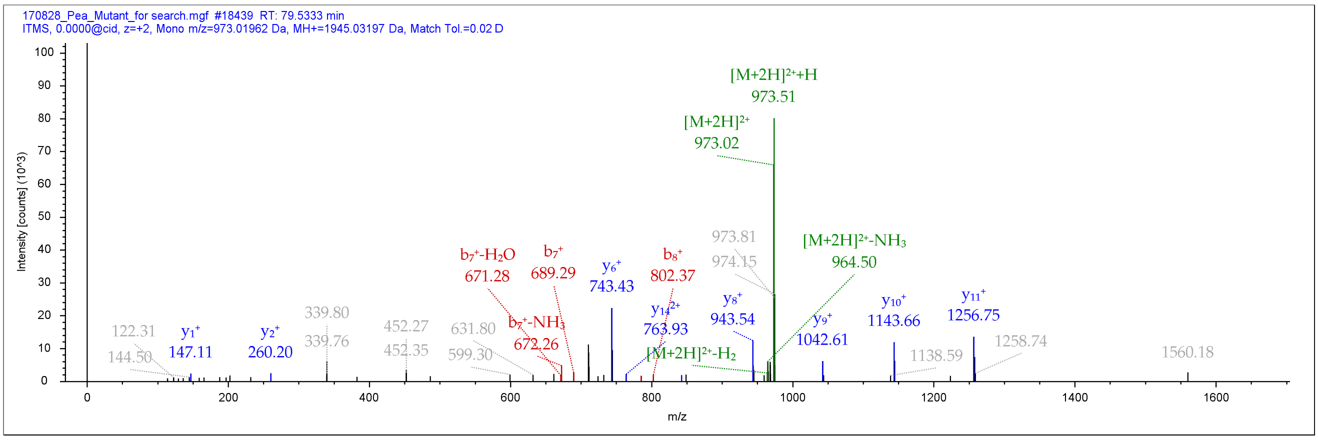

Figure S1-2.2 I3SCW0 (1 unique peptide(s), master protein)

$AAM_{Ox}ENGVLTVTVPKEEIK$, (1945.03197 Da, *m/z* 973.02, $[M+2H]^{2+}$), 3 PSM

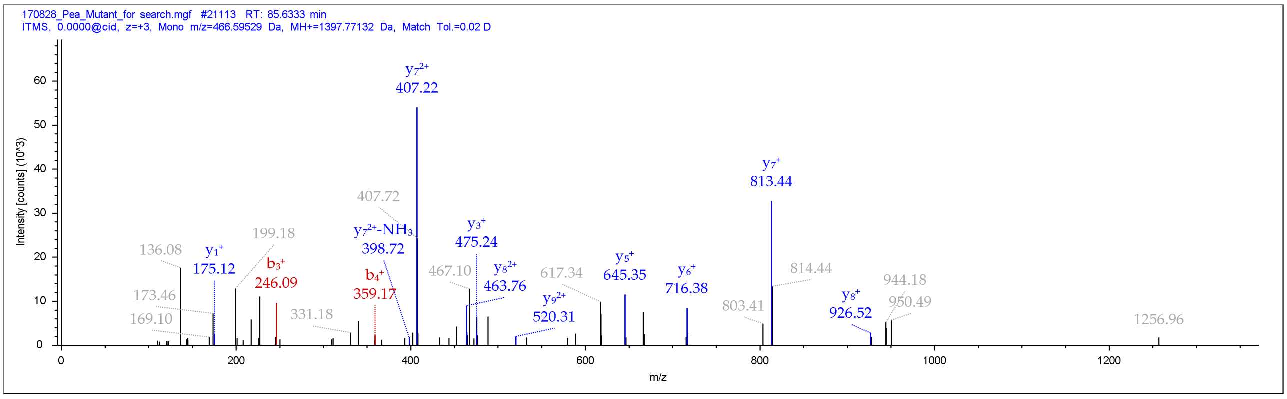

Figure S1-2.3 Medtr1g102870.1 (1 unique peptide(s), not master protein)

GGMIILPAGIYHR, (1397.77132 Da, *m/z* 466.60, $[M+3H]^{3+}$), 1 PSM

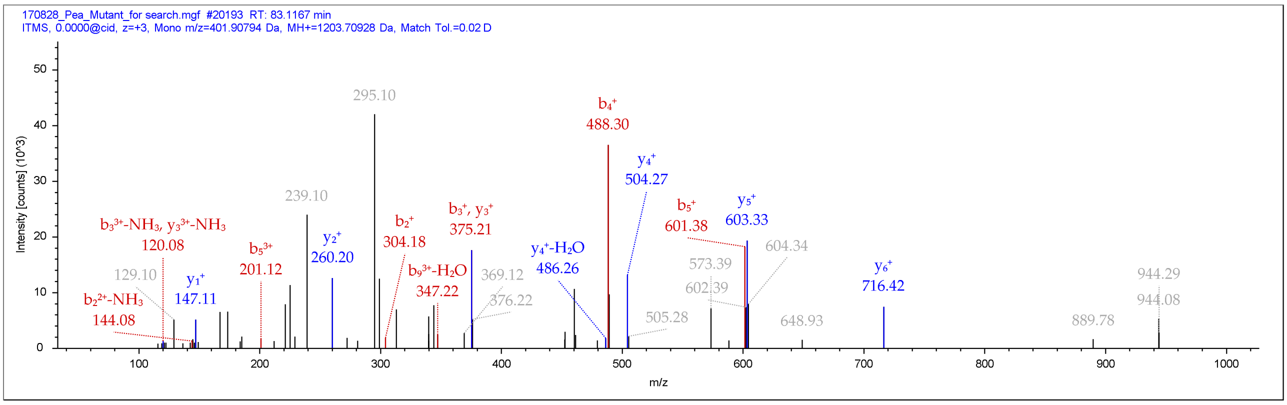

Figure S1-2.4 B7FH22 (1 unique peptide(s), master protein)

RFALLVEDLK, (1203.70928 Da, *m/z* 401.91, $[M+3H]^{3+}$), 3 PSM

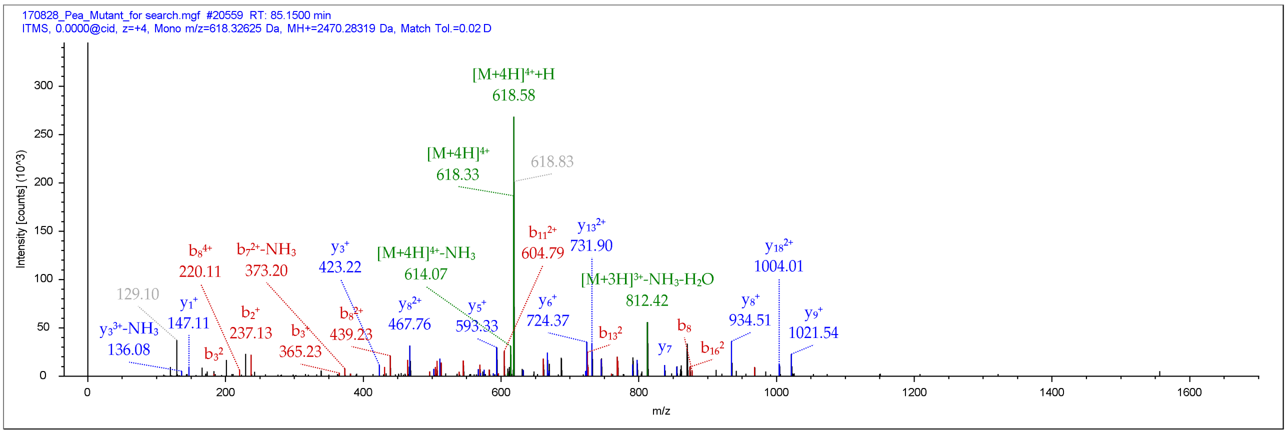

Figure S1-2.5 G7L8T3 (1 unique peptide(s), not master protein)

VHKVPSNDMEALKSPLMGIFEK, (2470.28319 Da, *m/z* 618.33, $[M+4H]^{4+}$), 16 PSM

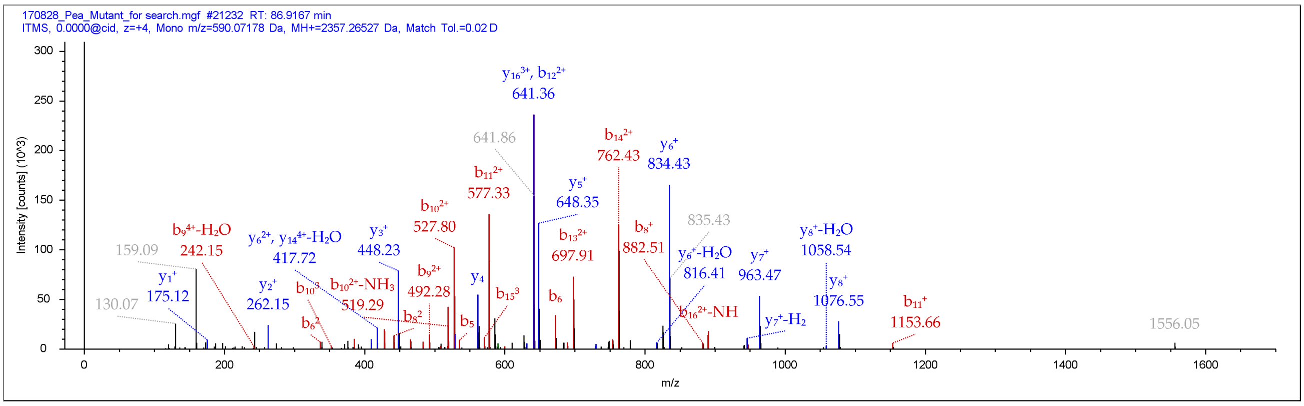

Figure S1-2.6 A2Q582 (1 unique peptide(s), not master protein)

RAHAVHPITAVQLEWSLWSR, (2357.26527 Da, *m/z* 590.07, $[M+4H]^{4+}$), 6 PSM

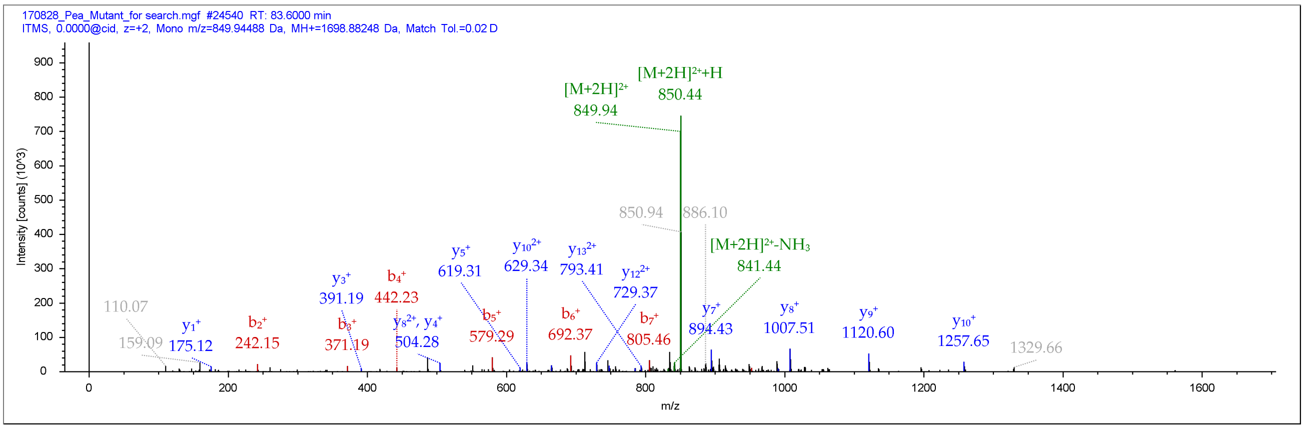

Figure S1-2.7 Q5QQ34 (1 unique peptide(s), master protein)

IQEAHLIFQDLSER, (1698.88248 Da, *m/z* 849.94, $[M+2H]^{2+}$), 2 PSM

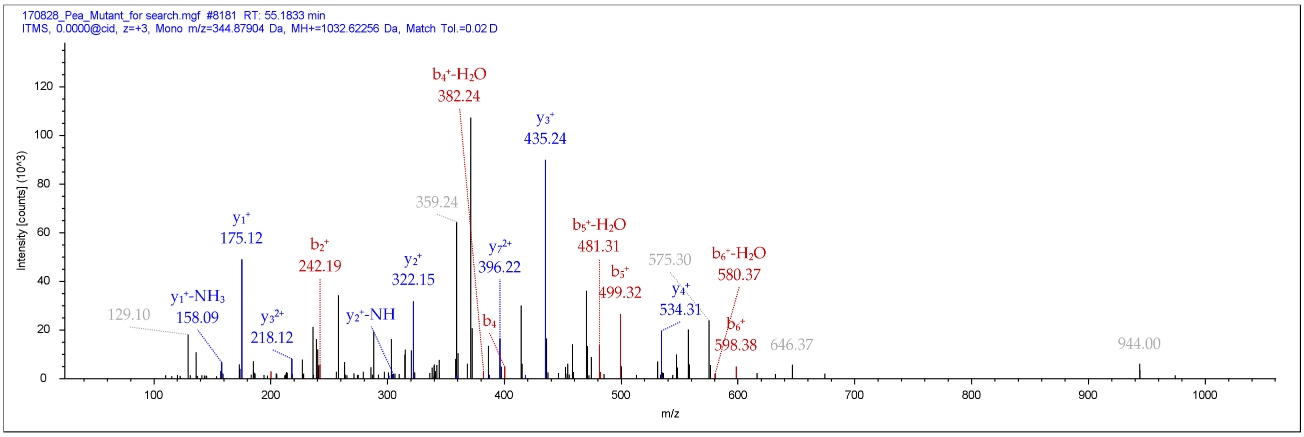

Figure S1-2.8 V8BZK0 (1 unique peptide(s), not master protein)

IKGTVVLM$_{Ox}$R, (1032.62256 Da, *m/z* 344.88, $[M+3H]^{3+}$), 3 PSM

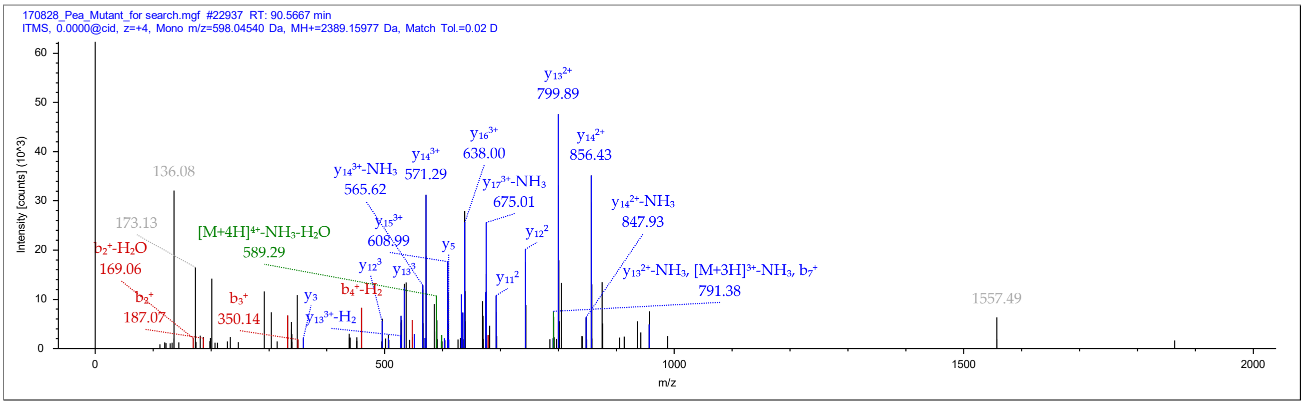

Figure S1-2.9 B7FJF0 (1 unique peptide(s), not master protein)

EGYQSLLNTDMERELNHLAR, (2389.15977 Da, *m/z* 598.05, $[M+4H]^{4+}$), 1 PSM

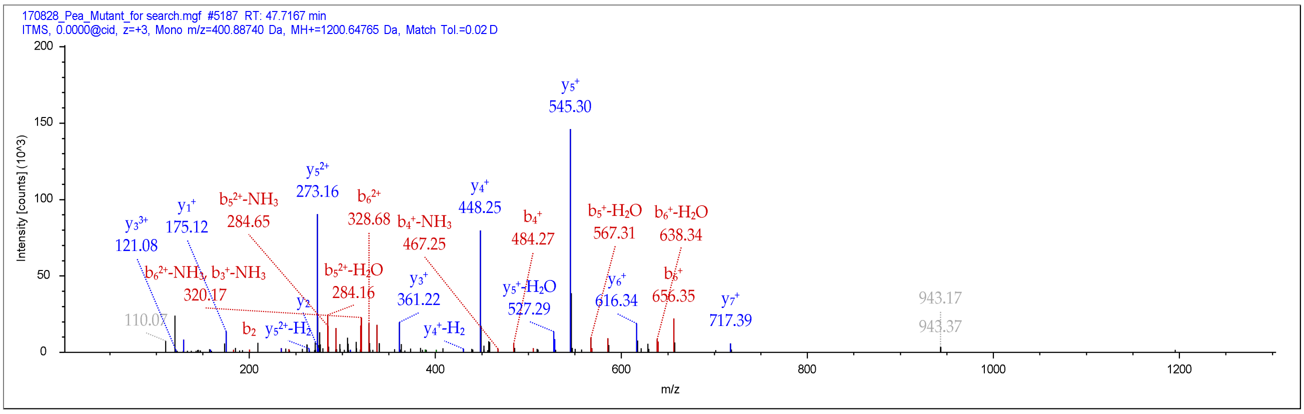

Figure S1-2.10 G7ILF2 (1 unique peptide(s), master protein)

KAHFTAPSSVR, (1200.64765 Da, *m/z* 400.89, $[M+3H]^{3+}$), 3 PSM

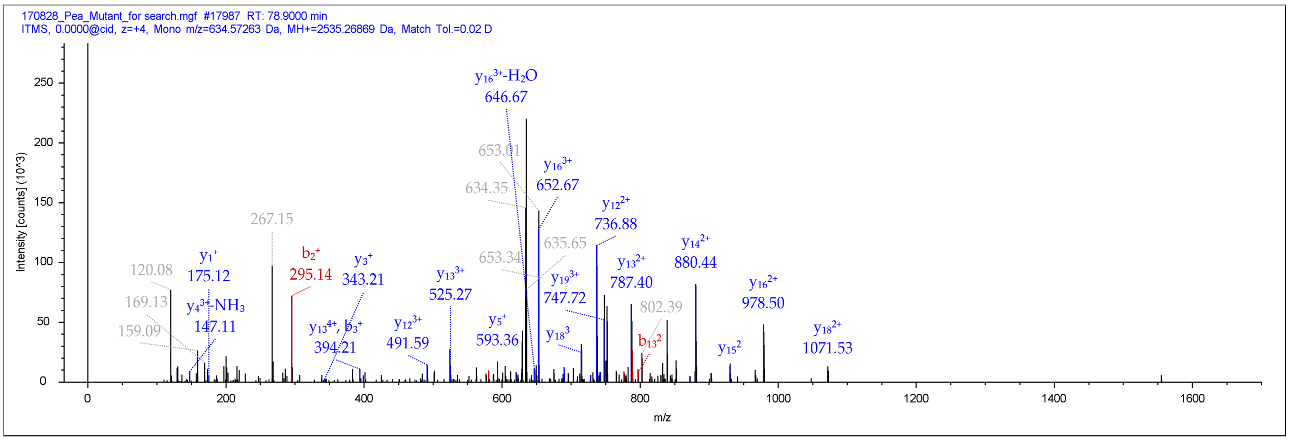

Figure S1-2.11 I3SYE6 (1 unique peptide(s), master protein)

FFVGEPVWTPYNRPHDHLPAR, (2535.26869 Da, *m/z* 634.57, $[M+4H]^{4+}$), 1 PSM

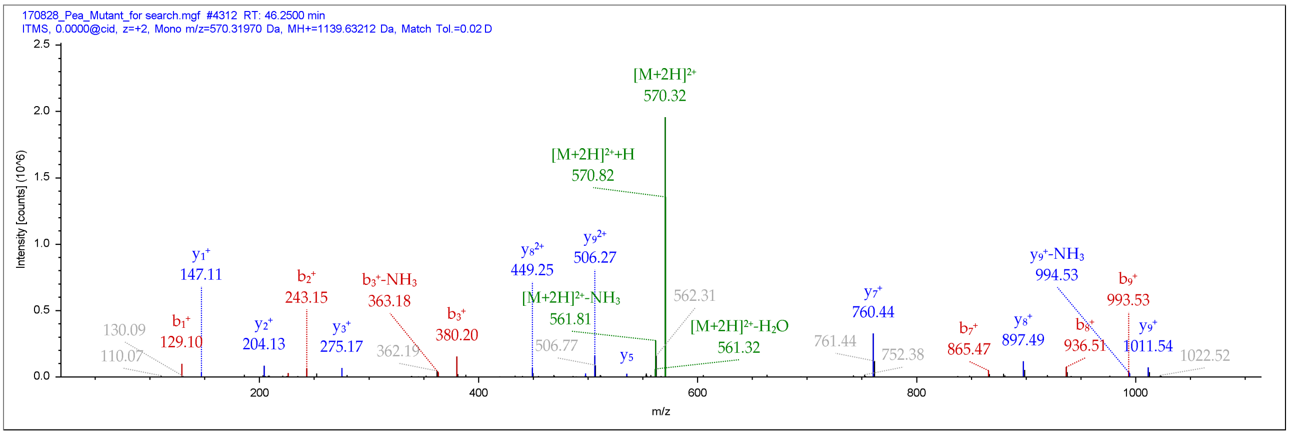

Figure S1-2.12 Medtr5g019780.1 (2 unique peptide(s), master protein)

KNHPQFLAGK, (1139.63212 Da, *m/z* 570.32, $[M+2H]^{2+}$), 6 PSM

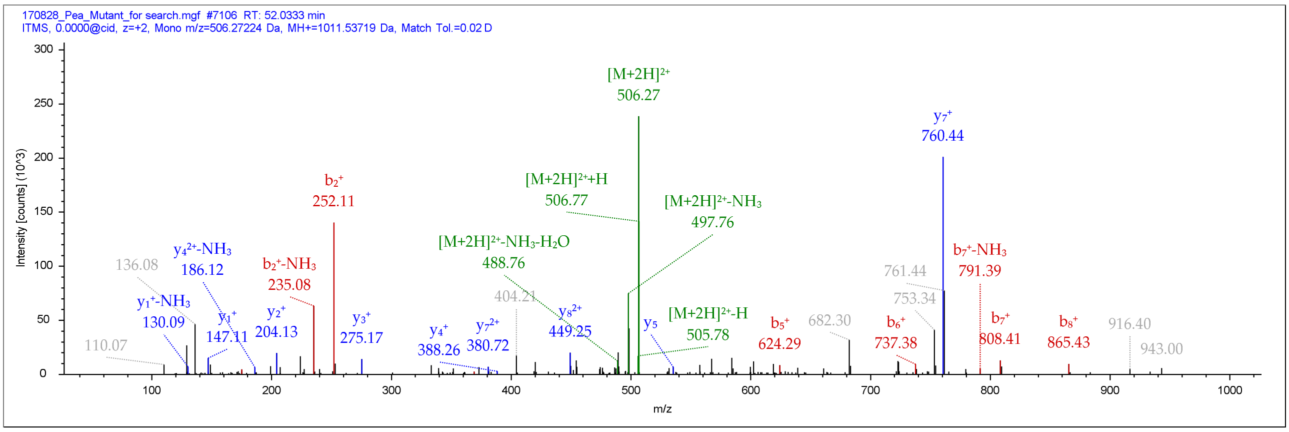

Figure S1-2.13 Medtr5g019780.1 (2 unique peptide(s), master protein)

NHPQFLAGK, (1011.53719 Da, *m/z* 506.27, $[M+2H]^{2+}$), 5 PSM

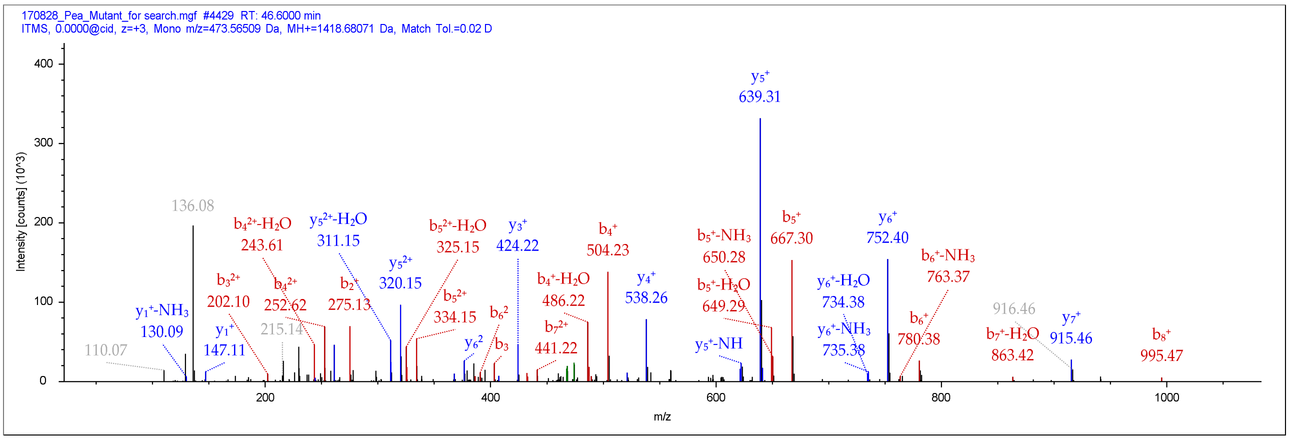

Figure S1-2.14 I3T560 (1 unique peptide(s), not master protein)

HHQTYITNYNK, (1418.68071 Da, *m/z* 473.56, $[M+3H]^{3+}$),7 PSM

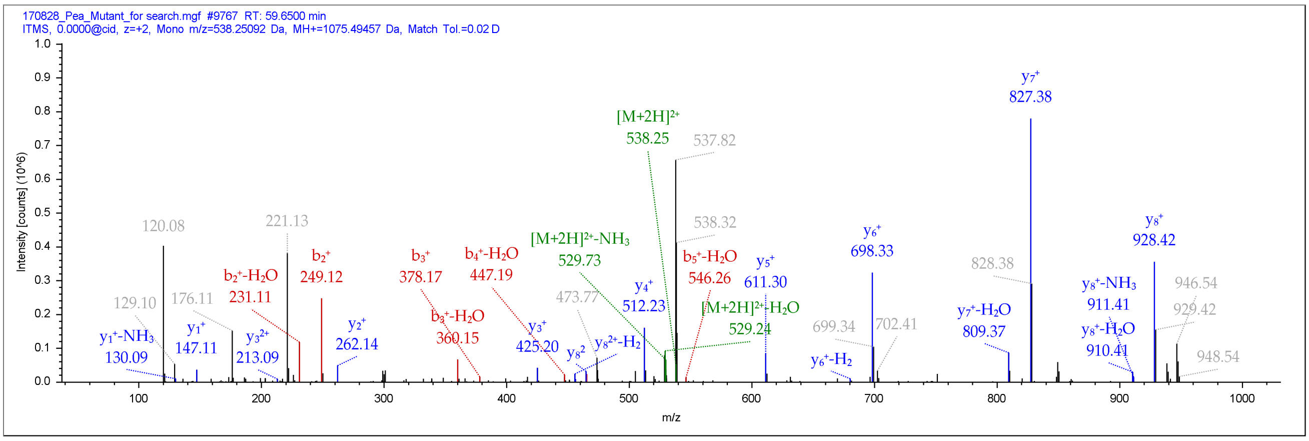

Figure S1-2.15 I3SU69 (1 unique peptide(s), master protein)

FTESVSYDK, (1075.49457 Da, *m/z* 538.25, $[M+2H]^{2+}$), 5 PSM

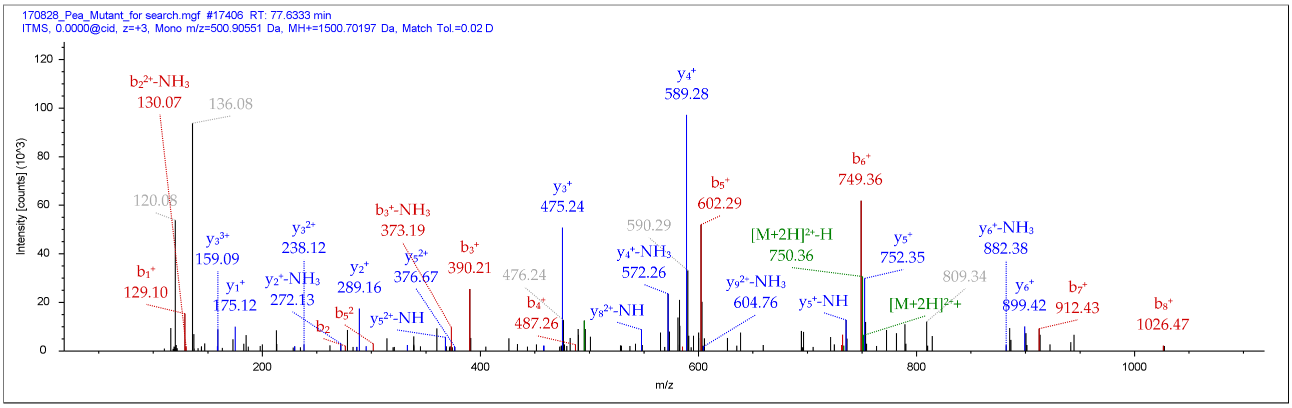

Figure S1-2.16 A0A072TSD1 (1 unique peptide(s), not master protein)

KFNPDFYNWNR, (1500.70197 Da, *m/z* 500.91, $[M+3H]^{3+}$), 5 PSM

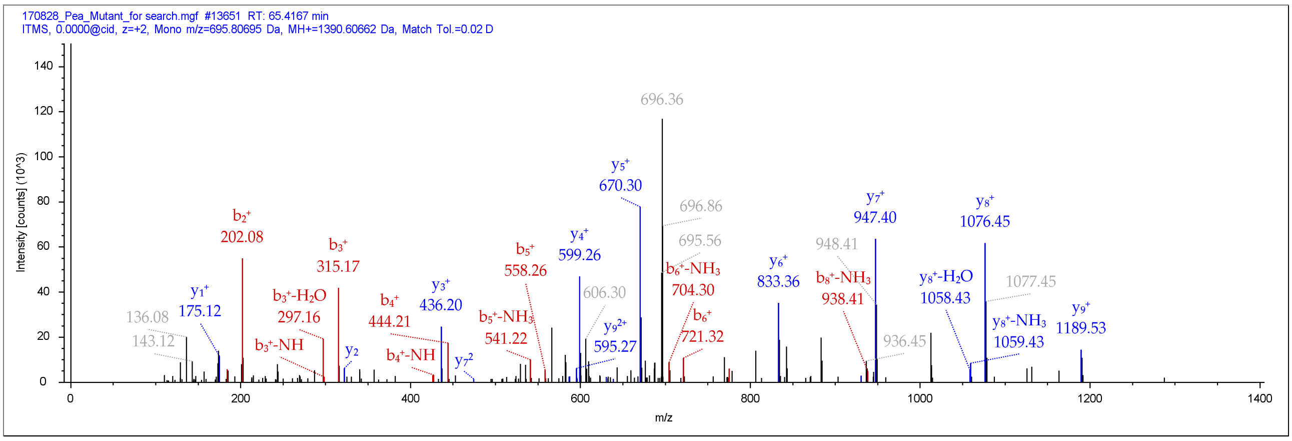

Figure S1-2.17 V7C9P5 (1 unique peptide(s), not master protein)

NSLENYAYNM$_{Ox}$R, (1390.60662 Da, *m/z* 695.81, $[M+2H]^{2+}$), 2 PSM

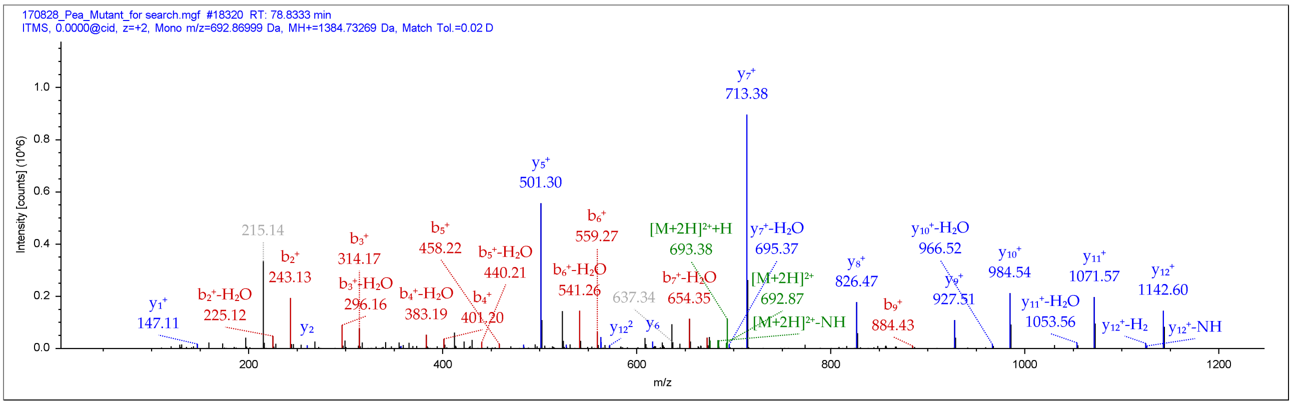

Figure S1-2.18 A0A072TMR3 (2 unique peptide(s) peptides, master protein)

EIASGTIPDPGSLK, (1384.73269 Da, *m/z* 692.87, $[M+2H]^{2+}$), 3 PSM

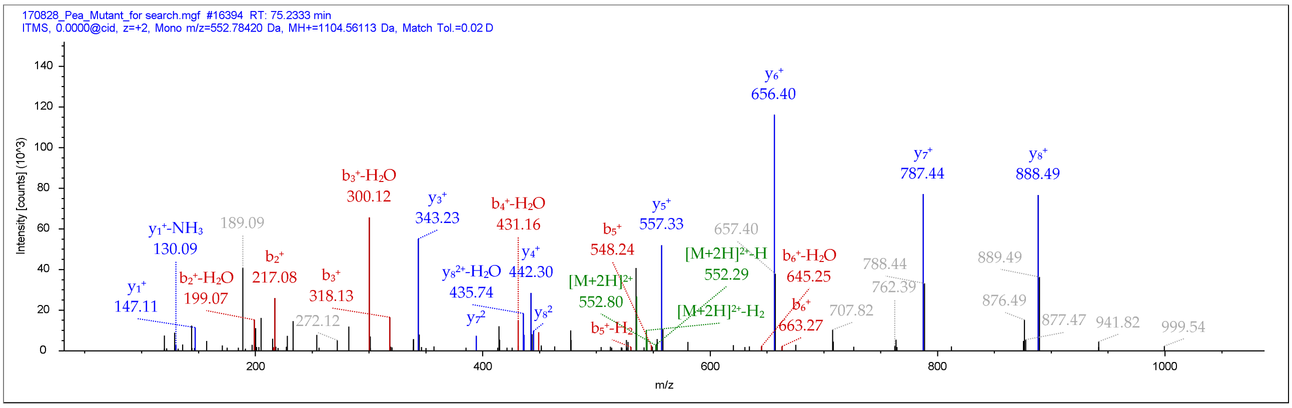

Figure S1-2.19 A0A072TMR3 (2 unique peptide(s) peptides, master protein)

DTTMVDVPVK, (1104.56113 Da, *m/z* 552.78, $[M+2H]^{2+}$), 3 PSM

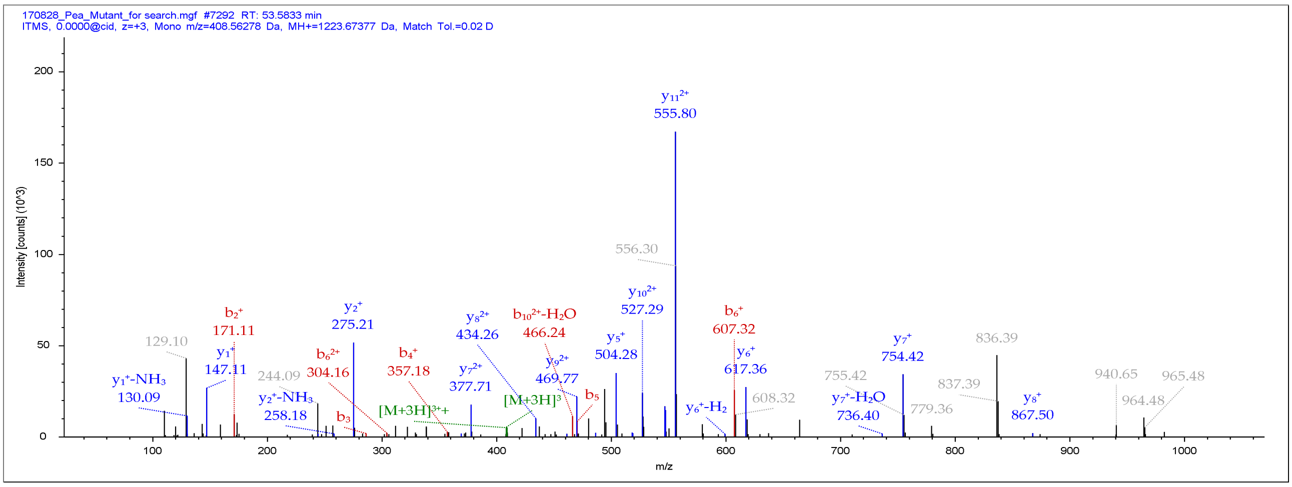

Figure S1-2.20 B1NY79 (1 unique peptide(s), master protein)

IGDALHIGGDKK, (1223.67377 Da, *m/z* 408.56, $[M+3H]^{3+}$), 1 PSM

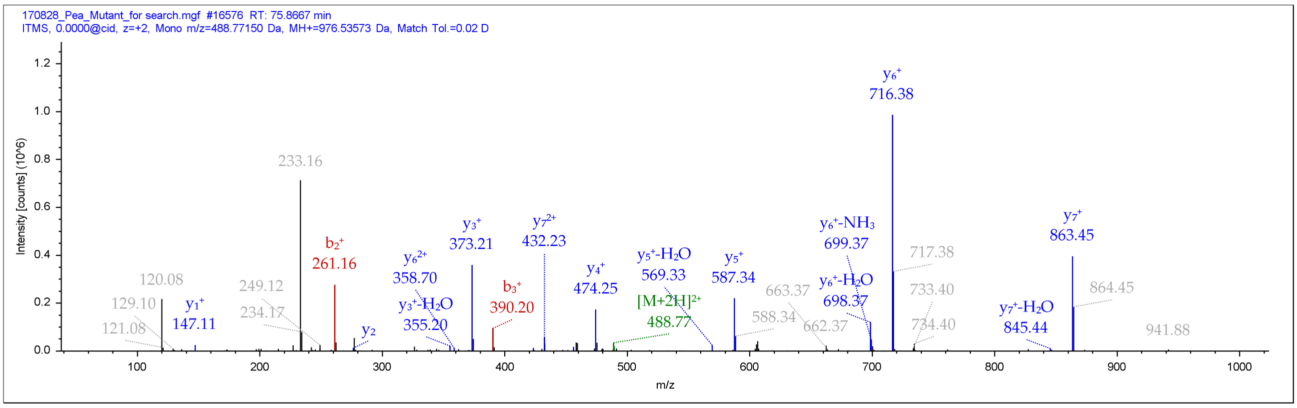

Figure S1-2.21 B5U8K3 (1 unique peptide(s), master protein)

LFEITPEK, (976.53573 Da, *m/z* 488.77, $[M+2H]^{2+}$), 16 PSM

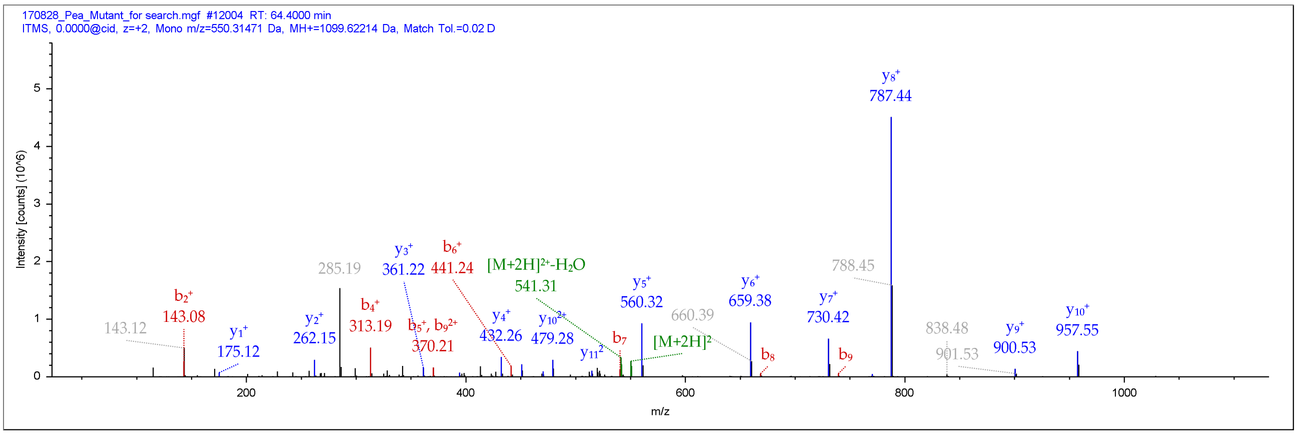

Figure S1-2.22 I3S2D8 (1 unique peptide(s), master protein)

AAGIGAVQAVSR, (1099.62214 Da, *m/z* 550.31, $[M+2H]^{2+}$), 2 PSM

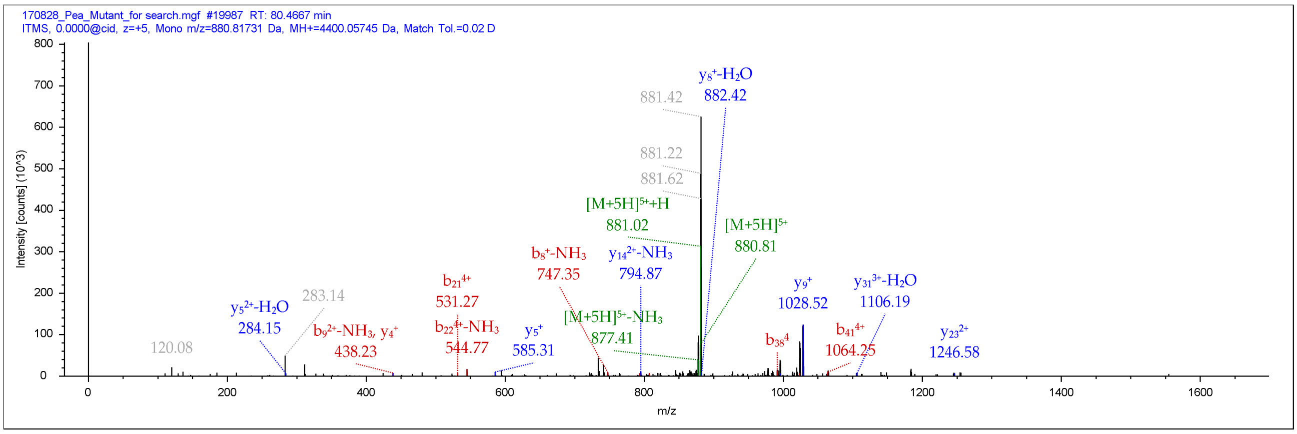

Figure S1-2.23 V8BVA1 (1 unique peptide(s), master protein)

ANSLAGSYKSSNSSWALSPGRAESPAMSVESM$_{Ox}$DKPMSFSGFK, (4400.05745 Da, *m/z* 880.82, $[M+5H]^{5+}$), 6 PSM

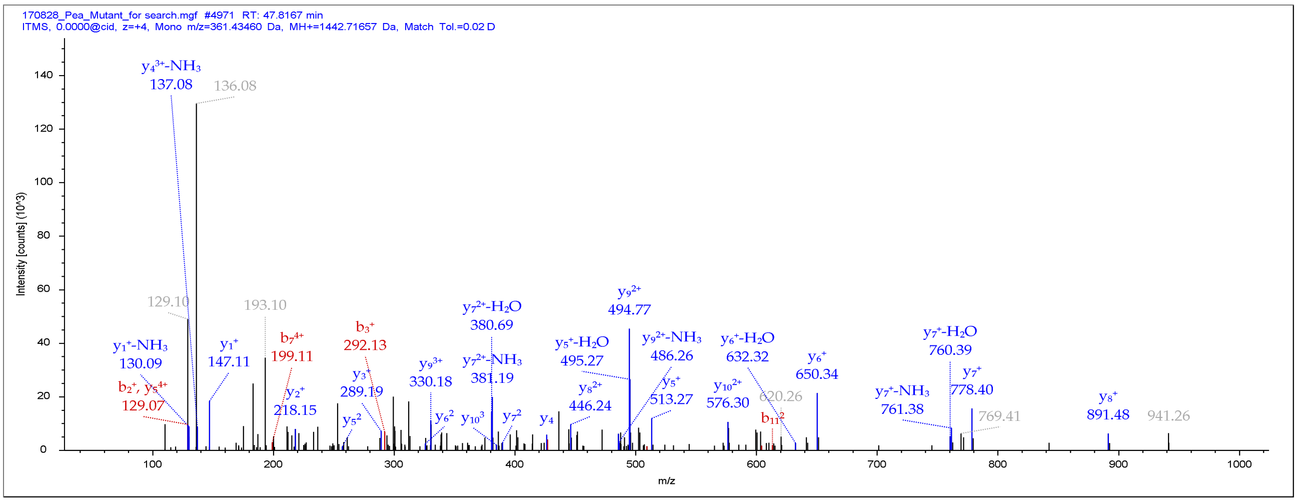

Figure S1-2.24 Medtr1g095155.1 (1 unique peptide(s), master protein)

AGYYPIQHSHAAK, (1442.71657 Da, *m/z* 361.43, $[M+4H]^{4+}$), 1 PSM

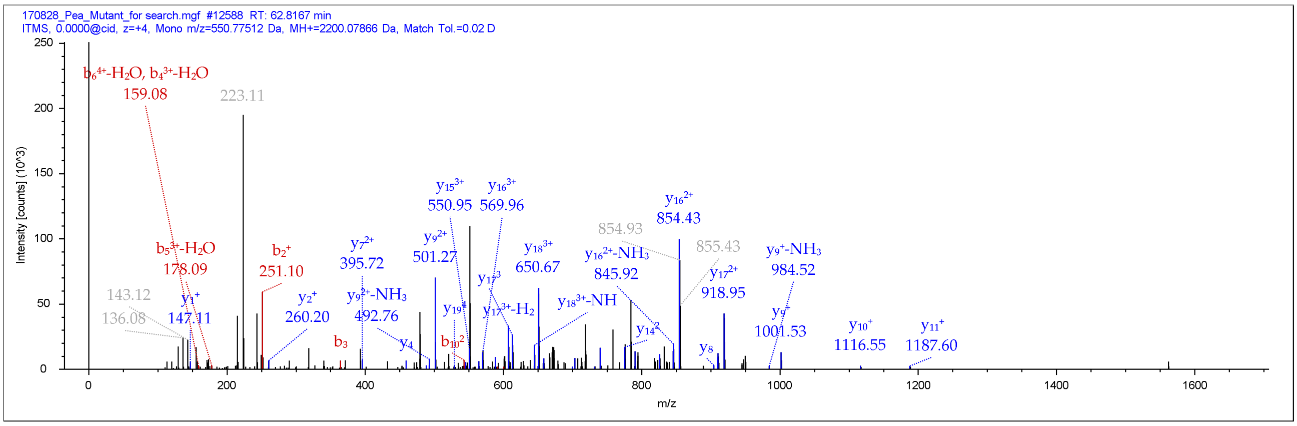

Figure S1-2.25 Q38JC8 (1 unique peptide(s), master protein)

SYIEGTAYKADPNSDEAKLK, (2200.07866 Da, *m/z* 550.78, $[M+4H]^{4+}$), 1 PSM

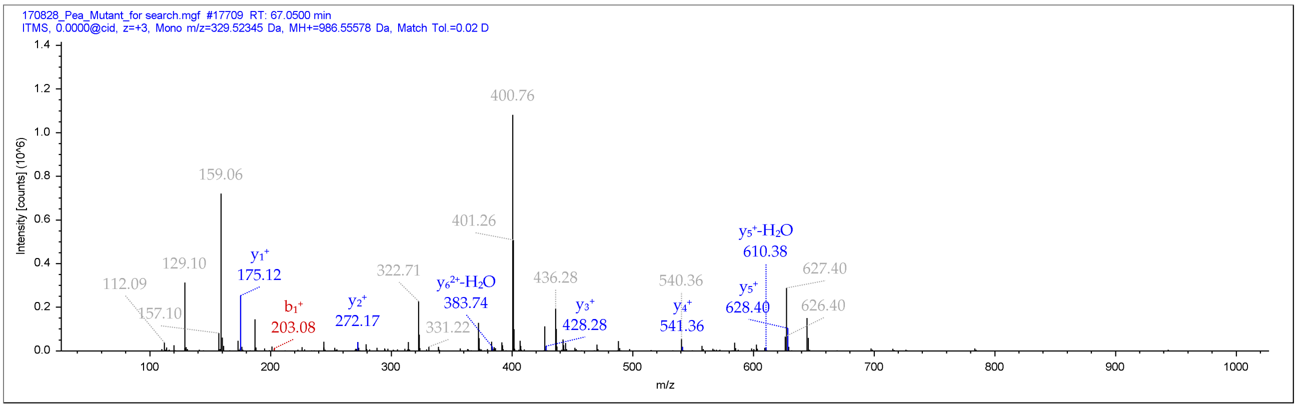

Figure S1-2.26 A0A072U7T5 (1 unique peptide(s), not master protein)

W_{Ox}RSLRPR, (986.55578 Da, *m/z* 329.52, $[M+3H]^{3+}$), 1 PSM

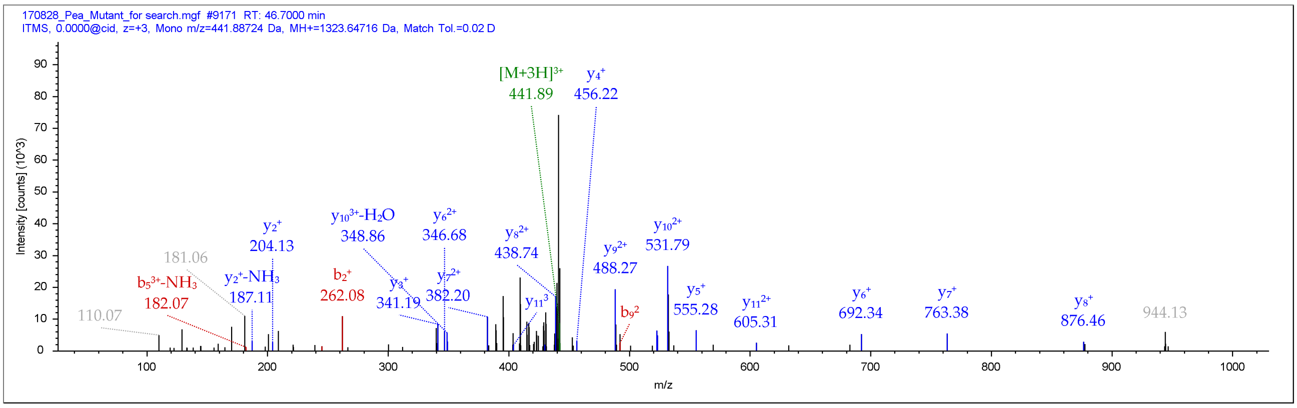

Figure S1-2.27 G7IH13 (1 unique peptide(s), not master protein)

NM$_{Ox}$SVIAHVDHGK, (1323.64716 Da, *m/z* 441.89, [M+3H]$^{3+}$), 5 PSM

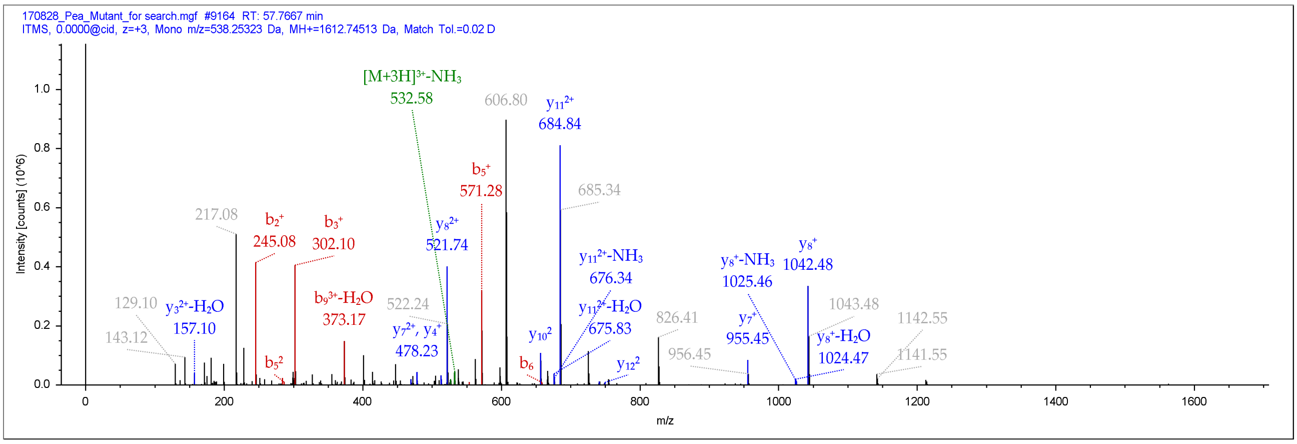

Figure S1-2.28 I3SRR2 (1 unique peptide(s), master protein)

DEGRISM$_{Ox}$KW$_{Ox}$M$_{Ox}$PSK, (1612.74513 Da, *m/z* 538.25, [M+3H]$^{3+}$), 2 PSM

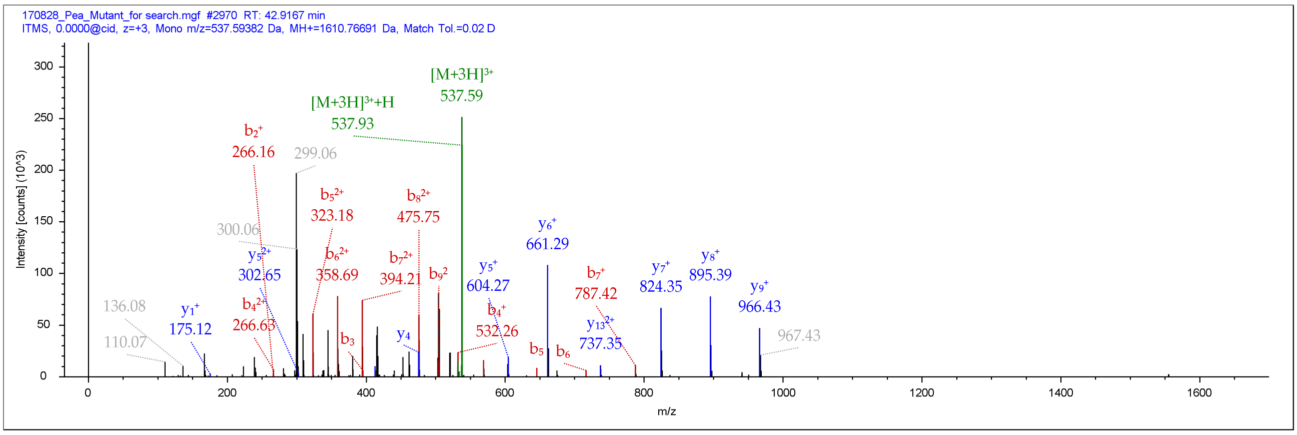

Figure S1-2.29 G7KG34 (2 unique peptide(s), master protein)

HKEHIAAYGEGNER, (1610.76691 Da, *m/z* 537.59, [M+3H]$^{3+}$), 3 PSM

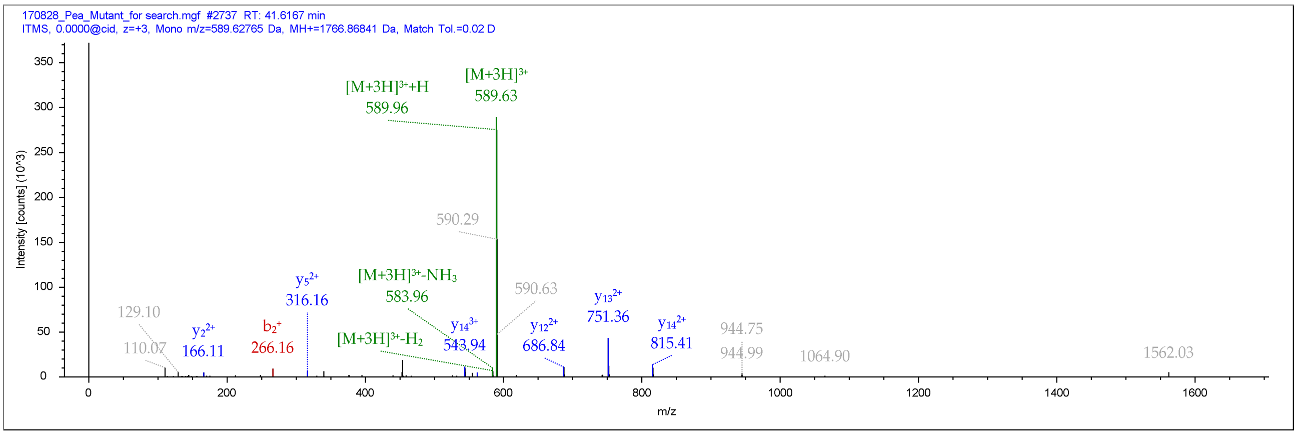

Figure S1-2.30 G7KG34 (2 unique peptide(s), master protein)

HKEHIAAYGEGNERR, (1766.86841 Da, *m/z* 589.63, $[M+3H]^{3+}$), 2 PSM

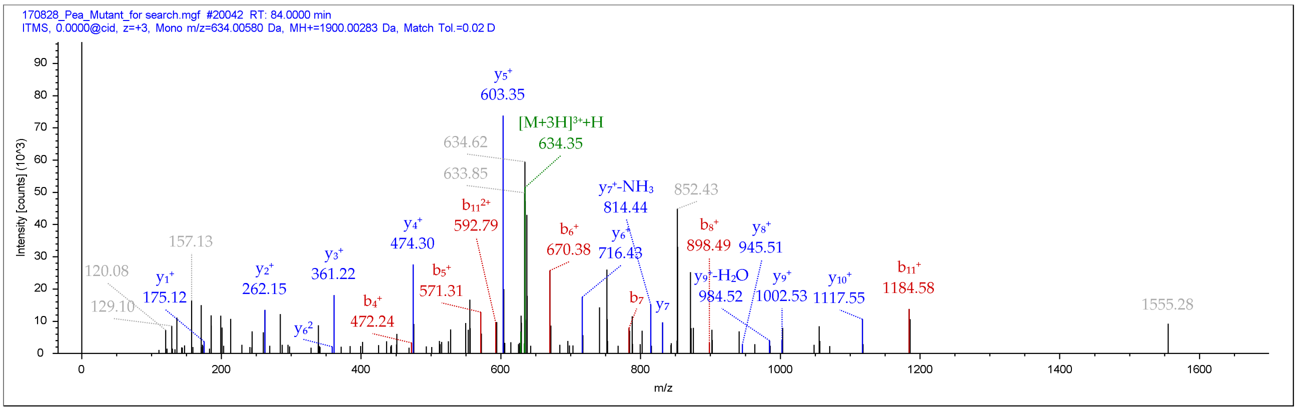

Figure S1-2.31 B7FN14 (1 unique peptide(s), master protein)

VKDEVVLDGNDIELVSR, (1900.00283 Da, *m/z* 634.01, $[M+3H]^{3+}$), 6 PSM

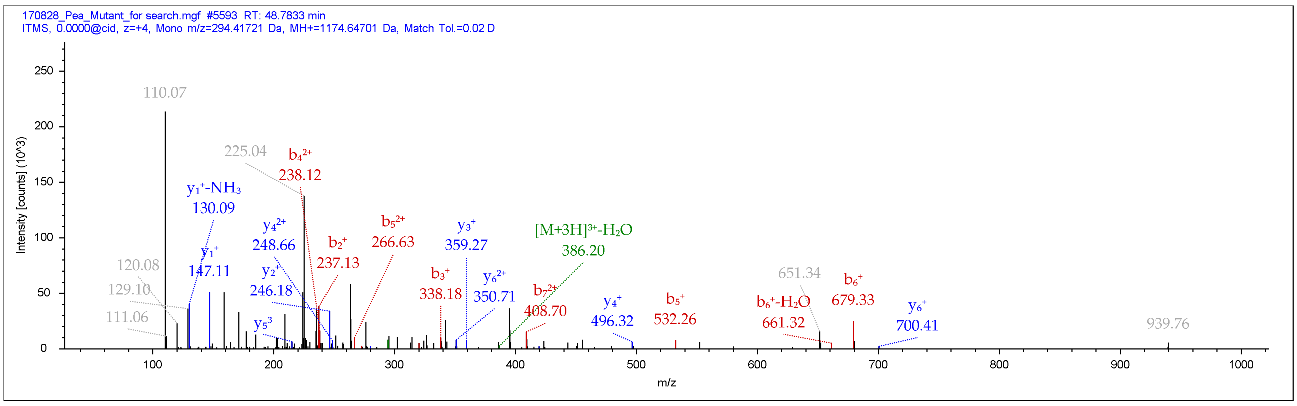

Figure S1-2.32 A0A072TQ47 (1 unique peptide(s) peptide, not master protein)

HVTHGFHLVK, (1174.64701 Da, *m/z* 294.42, $[M+4H]^{4+}$), 2 PSM

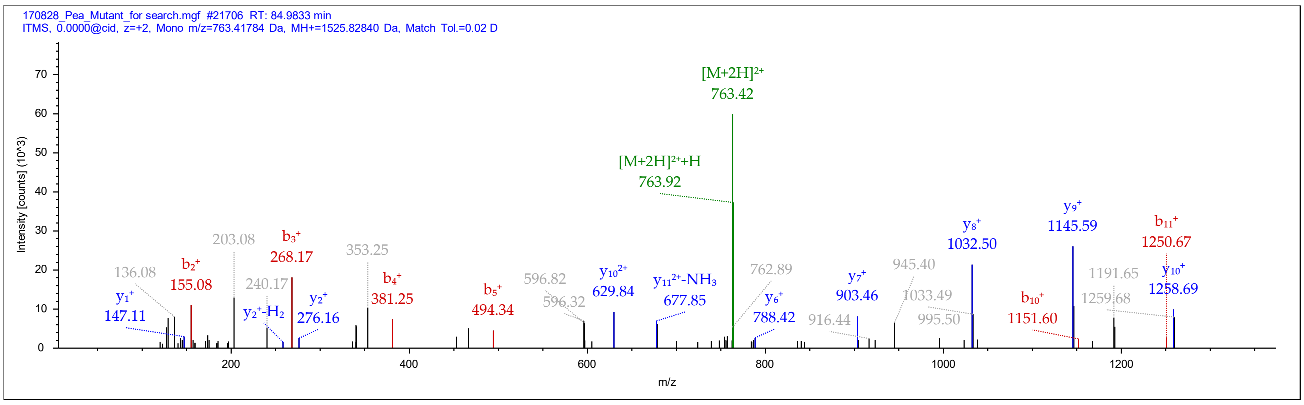

Figure S1-2.33 A0PG70 (1 unique peptide(s), master protein)

GPILLEDYHLVEK, (1525.82840 Da, *m/z* 763.42, $[M+2H]^{2+}$), 2 PSM

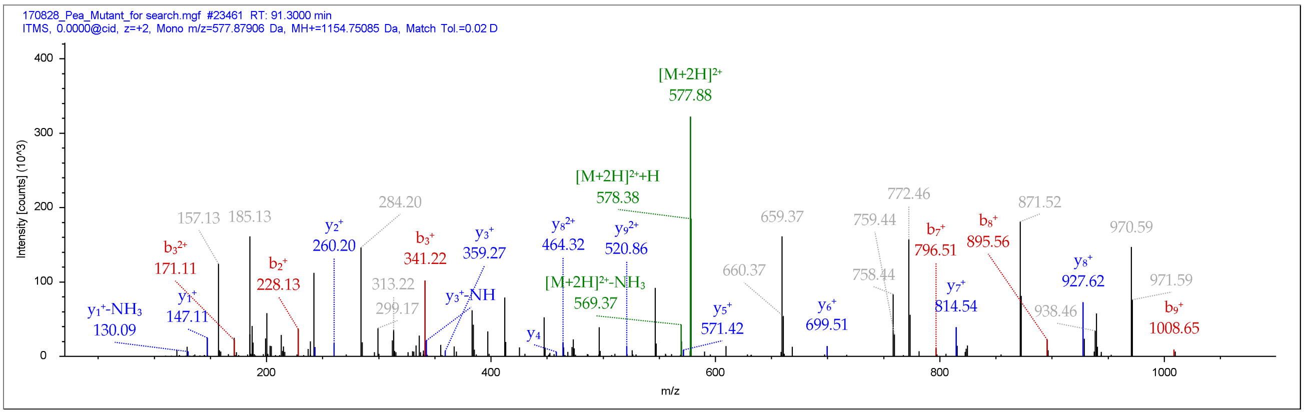

Figure S1-2.34 A0A072VE37 (1 unique peptide(s), not master protein)

NLLDKLVVLK, (1154.75085 Da, *m/z* 577.88, $[M+2H]^{2+}$), 12 PSM

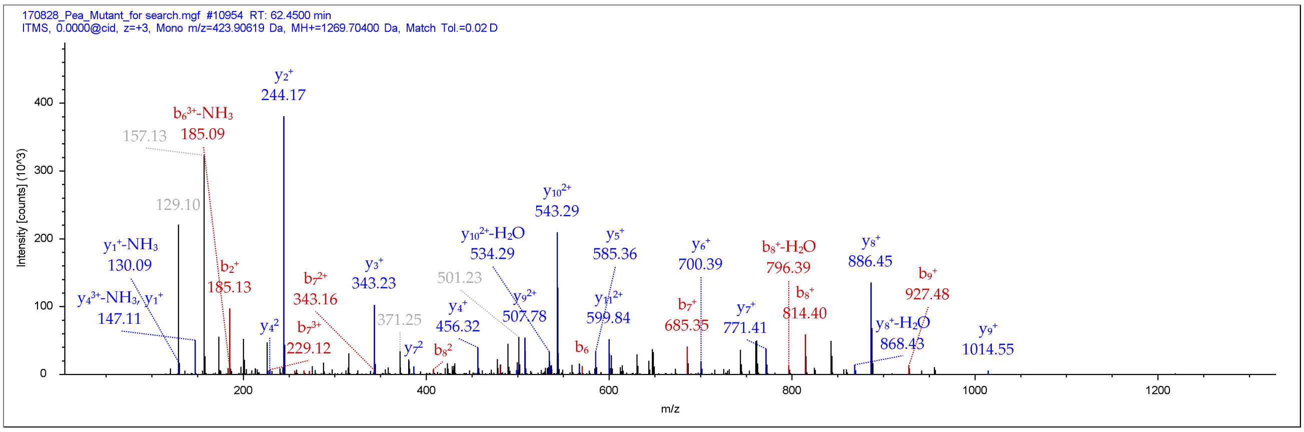

Figure S1-2.35 G7IMZ3 (1 unique peptide(s), master protein)

ALAKDADEIVPK, (1269.70400 Da, *m/z* 423.91, $[M+3H]^{3+}$), 13 PSM

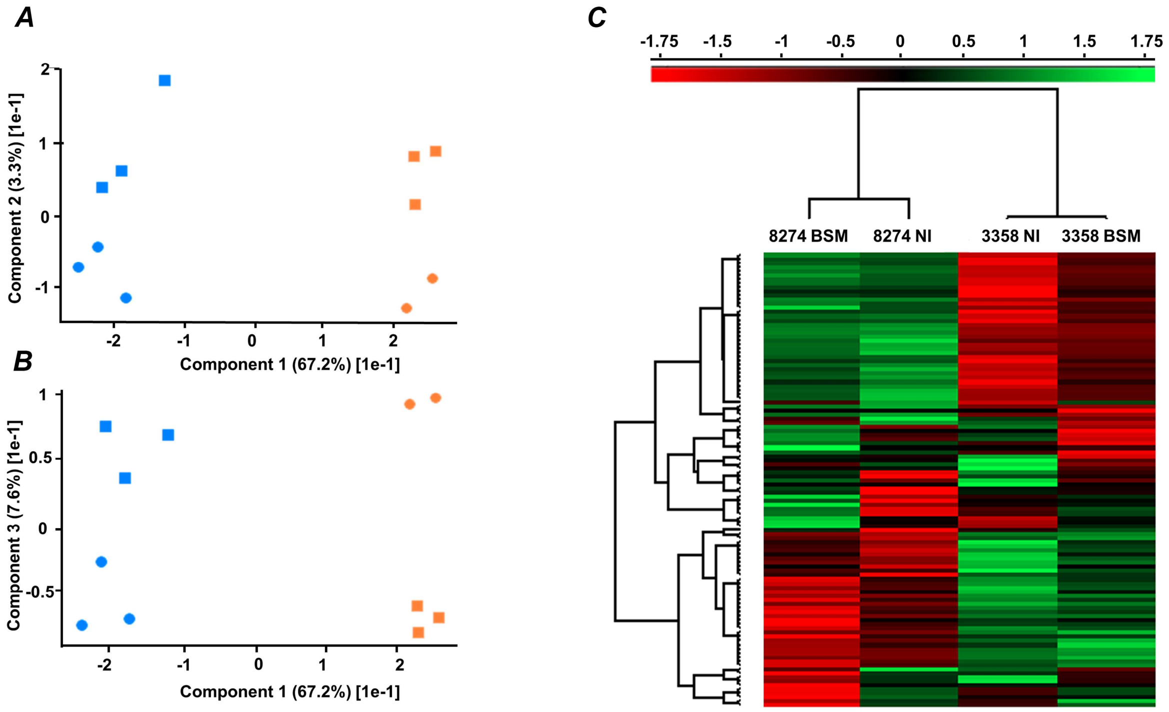

Figure S1-3 Post-processing of the label-free quantification data with Perseus 1.6.0.0 software. The raw data were acquired by nanoHPLC-ESI-Q-Orbitrap-MS using a data-dependent acquisition algorithm, whereas processing relied on Progenesis QI software. The samples (tryptic digests) were obtained from the seeds of pea (*P. sativum* L) plants, lines K-8274 (high EIBSM, A) and K-3358 (low EIBSM, B), grown with (BSM, beneficial soil microorganisms) and without (NI, not inoculated) simultaneous colonization of pea roots with rhizobia and arbuscular mycorrhizae (AM) fungi *R. irregularis*. The K-8274 (orange) and K-3358 (blue) pea lines could be separated by the first component (A,B), whereas BSM (squares) and NI (circles) were separated by the second (A) and third (B) components. Hierarchical clustering was done for average group values, calculated by three biological replicates (but only two replicates in NI group of line K-8274) (C). For the clustering of columns and rows Pearson and Spearman correlation coefficients were used as a distance measure.

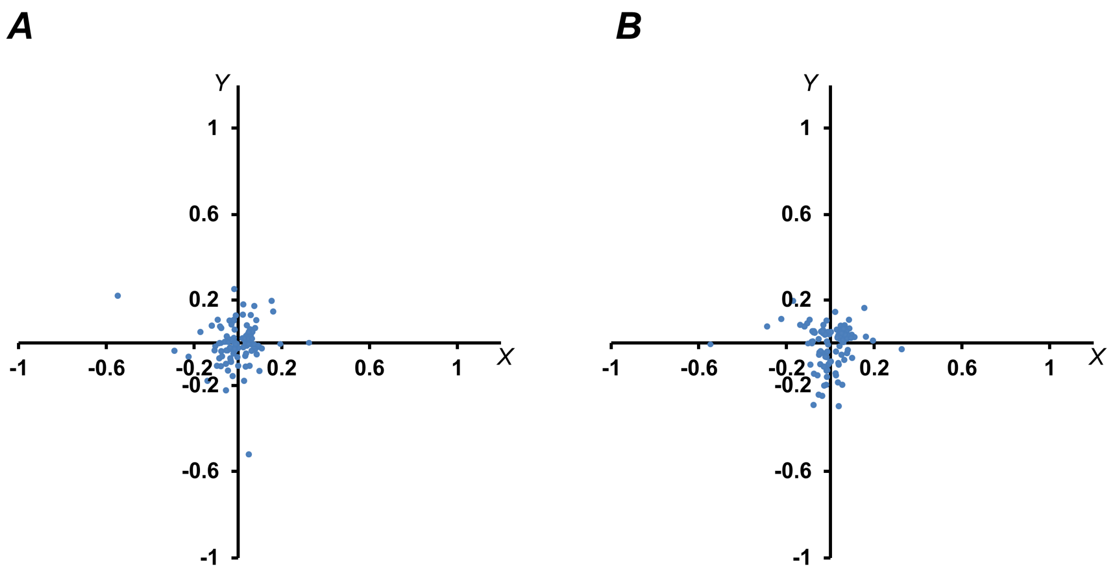

Figure S1-4 Loading plots for the principal component analysis of proteins obtained from the seeds of pea (*P. sativum* L) plants, lines K-8274 (high EIBSM) and K-3358 (low EIBSM). X axis denote loadings of the 1st component on both panels while Y axis denote 2nd component on panel A and 3rd component on panel B. Analysis was done in the Perseus 1.6.0.0 software.

Figure S1-5 Mass spectra and extracted ion chromatograms (left and right panels, respectively) obtained Progenesis QIP 4.0 software upon quantification procedure. For all but Figure S1-5.4 plots were obtained from spectra file of the 3rd replicate of the line K-8274; for S1-5.4 plots are obtained from spectra file of the 3rd replicate of the line K-3358.

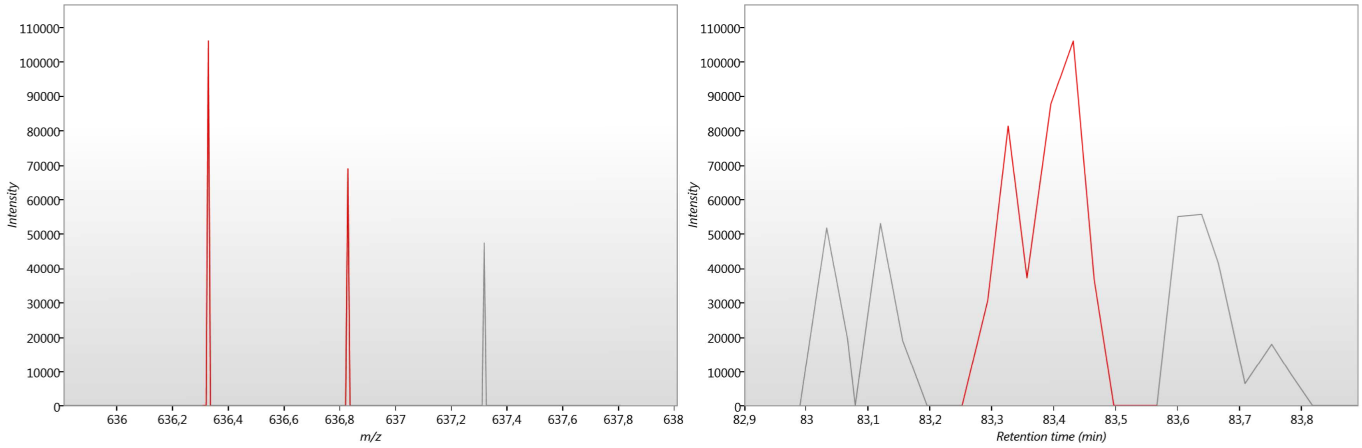

Figure S1-5.1 A0A072UGB7
VDSELIYAYAK, (t_R 83.43 min, *m/z* 636.32, $[M+2H]^{2+}$)

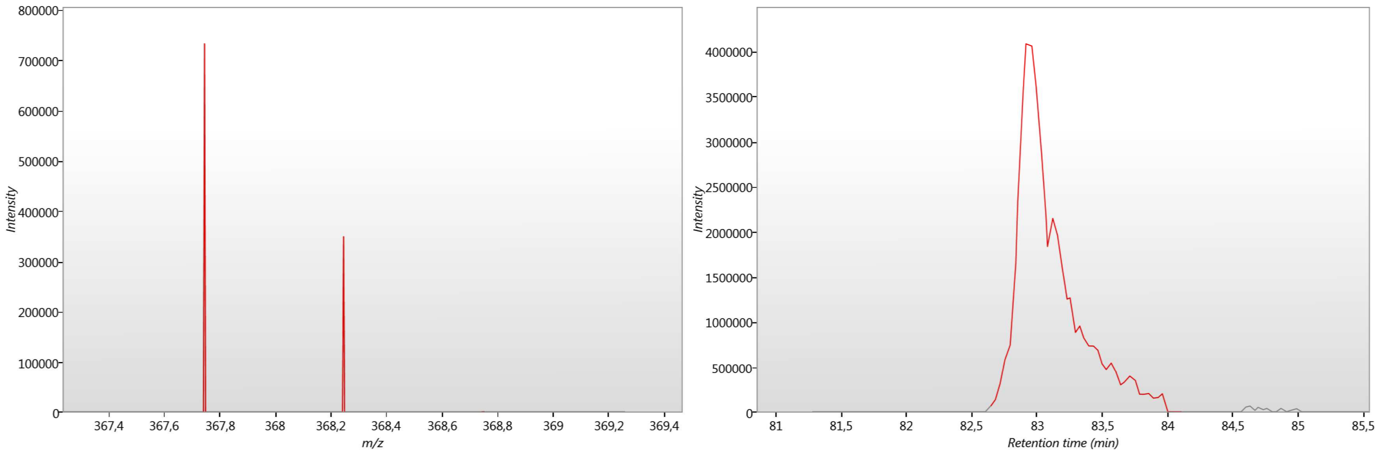

Figure S1-5.2 Lj3g3v0324640.1
TILFLK, (t_R 83.40 min, *m/z* 367.74, $[M+2H]^{2+}$)

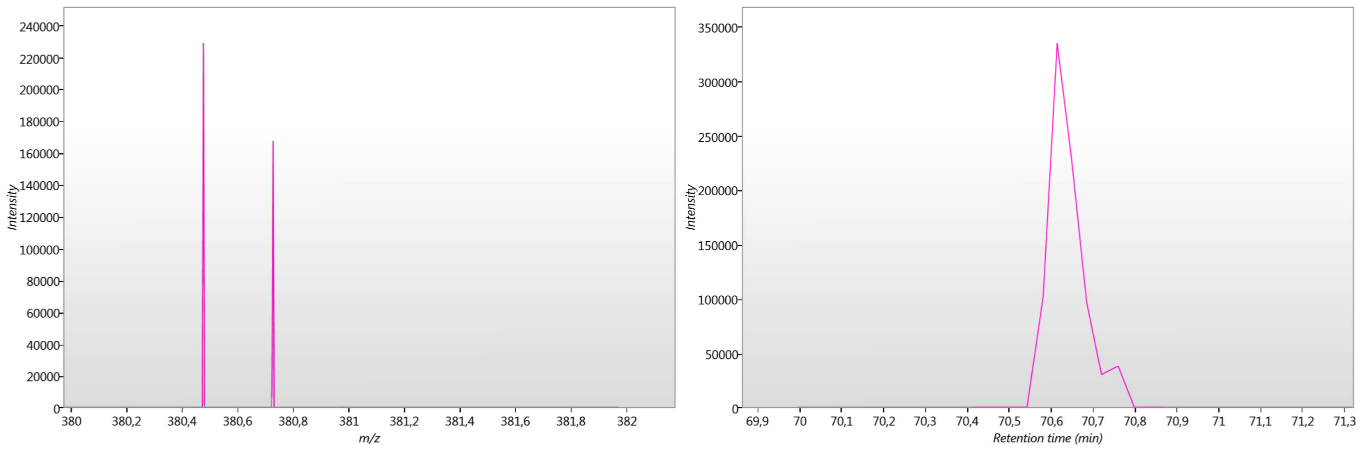

Figure S1-5.3 A0A072VJU4
SPLLLQMNPIHKK, (t_R 70.65 min, *m/z* 380.48, $[M+4H]^{4+}$)

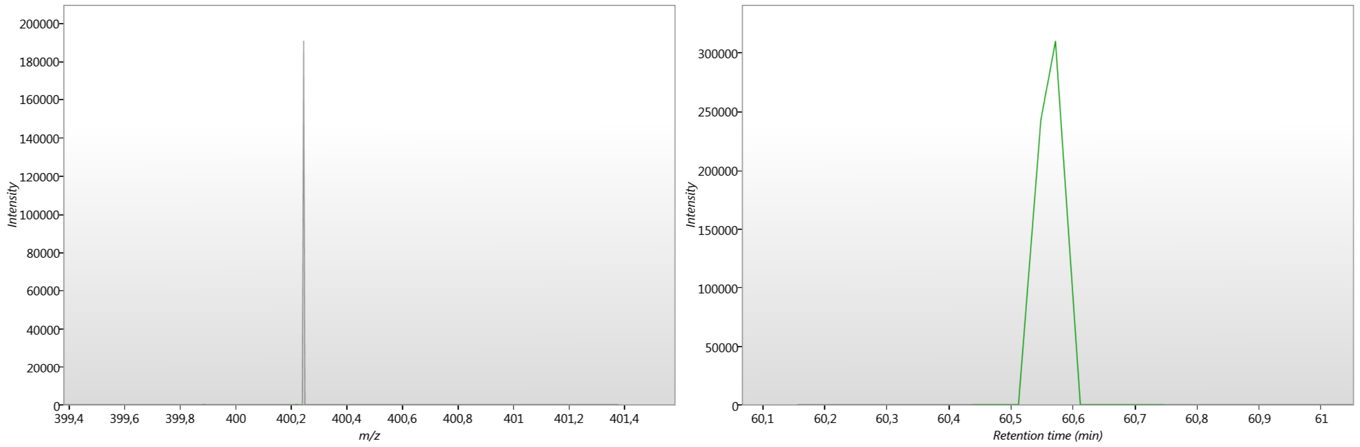

Figure S1-5.4 A0A072TYG8
APWSIQDKPR, (t_R 60.61 min, *m/z* 399.88, $[M+3H]^{3+}$)

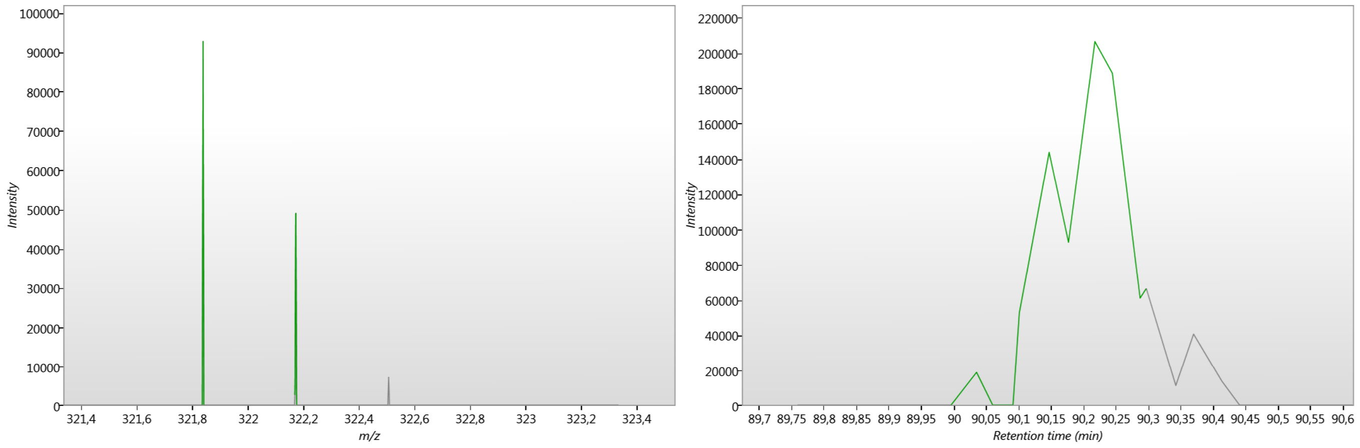

Figure S1-5.5 Lj0g3v0098069.1
NFIYFIF, (t_R 90.18 min, *m/z* 321.84, $[M+3H]^{3+}$)

References

1. Huang, Y.; Niu, B.; Gao, Y.; Fu, L.; Li, W. CD-HIT Suite : a web server for clustering and comparing biological sequences. *Bioinformatics* **2010**, *26*, 680–682.
